# The *Compass-like* Locus, Exclusive to the Ambulacrarians, Encodes a Chromatin Insulator Binding Protein in the Sea Urchin Embryo

**DOI:** 10.1371/journal.pgen.1003847

**Published:** 2013-09-26

**Authors:** Vincenzo Cavalieri, Raffaella Melfi, Giovanni Spinelli

**Affiliations:** Dipartimento di Scienze e Tecnologie Biologiche, Chimiche e Farmaceutiche, Università di Palermo, Palermo, Italy; The University of North Carolina at Chapel Hill, United States of America

## Abstract

Chromatin insulators are eukaryotic genome elements that upon binding of specific proteins display barrier and/or enhancer-blocking activity. Although several insulators have been described throughout various metazoans, much less is known about proteins that mediate their functions. This article deals with the identification and functional characterization in *Paracentrotus lividus* of COMPASS-like (CMPl), a novel echinoderm insulator binding protein. Phylogenetic analysis shows that the CMPl factor, encoded by the alternative spliced *Cmp*/*Cmpl* transcript, is the founder of a novel ambulacrarian-specific family of Homeodomain proteins containing the Compass domain. Specific association of CMPl with the *boxB cis*-element of the *sns5* chromatin insulator is demonstrated by using a yeast one-hybrid system, and further corroborated by ChIP-qPCR and *trans*-activation assays in developing sea urchin embryos. The *sns5* insulator lies within the early histone gene cluster, basically between the *H2A* enhancer and *H1* promoter. To assess the functional role of CMPl within this locus, we challenged the activity of CMPl by two distinct experimental strategies. First we expressed in the developing embryo a chimeric protein, containing the DNA-binding domain of CMPl, which efficiently compete with the endogenous CMPl for the binding to the *boxB* sequence. Second, to titrate the embryonic CMPl protein, we microinjected an affinity-purified CMPl antibody. In both the experimental assays we congruently observed the loss of the enhancer-blocking function of *sns5*, as indicated by the specific increase of the *H1* expression level. Furthermore, microinjection of the CMPl antiserum in combination with a synthetic mRNA encoding a forced repressor of the *H2A* enhancer-bound MBF1 factor restores the normal *H1* mRNA abundance. Altogether, these results strongly support the conclusion that the recruitment of CMPl on *sns5* is required for buffering the *H1* promoter from the *H2A* enhancer activity, and this, in turn, accounts for the different level of accumulation of early linker and nucleosomal transcripts.

## Author Summary

Mounting evidence in several model organisms collectively demonstrates a role for the DNA-protein complexes known as chromatin insulators in orchestrating the functional domain organization of the eukaryotic genome. Several DNA elements displaying features of insulators, *viz* barrier and/or directional enhancer-blocking activity, have been identified in yeast, *Drosophila*, sea urchin, vertebrates and plants; however, proteins that bind these DNA sequences eliciting insulator activities are far less known. Here we identify a novel protein, COMPASS-like (CMPl), which is expressed exclusively by the ambulacrarian group of metazoans and interacts directly with the sea urchin *sns5* insulator. *Sns5* lies within the early histone gene cluster, basically between the *H2A* enhancer and *H1* promoter, where it acts buffering the *H1* promoter from the *H2A* enhancer influence. Intriguingly, we find that CMPl role is absolutely required for the *sns5* activity, therefore imposing the different level of accumulation of the linker and nucleosomal transcripts. Overall, our findings add an interesting and novel facet to the chromatin insulator field, highlighting the surprisingly low evolutionary conservation of *trans*-acting factors binding to chromatin insulators. This opens the possibility that multiple lineage-specific factors modulate chromatin organization in different metazoans.

## Introduction

Chromatin insulators are specialized DNA elements that upon binding of specific proteins display barrier and/or directional enhancer-blocking activity. The analysis of the genome-wide localization of insulator binding proteins (IBPs) in vertebrates and *Drosophila* suggests that insulators partition the eukaryotic genome in autonomous functional domains by promoting the formation of physical loop structures and/or mediate tethering of the chromatin fiber to structural elements within the nucleus [1], [2]. In vertebrates, CCCTC-binding factor (CTCF) is the only IBP that has been well characterized. Mechanistically, CTCF and its associated co-factors, most notably cohesin, are important in establishing long range chromatin interaction [3], [4]. This is illustrated by the CTCF-dependent intra- and inter-chromosomal interaction necessary for allele specific transcription within the mouse *β-globin* locus and at the imprinting control region in the *H19*/*Igf2* locus [5]–[7]. Similarly, upon binding near the *ins* and *syt8* promoters, located more that 300 kb away, CTCF stabilizes their interaction and affects gene expression at the human insulin locus [8].

Distinct families of insulators, defined by the IBPs necessary for their activity, have been described in *drosophila*. The best characterized IBPs are Zeste-white5 (Zw5) and Boundary Element Associated Factor 32 (BEAF-32), that bind to the first identified enhancer-blocking insulators *scs* and *scs′* [9], [10], Suppressor of Hair-wing [Su(Hw)] of the *gypsy* retrotransposon [11], and dCTCF [12]. The functions of all *Drosophila* insulators converge as chromatin organizer into that of CTCF in vertebrates. Zw5 and BEAF-32 interact with each other to generate a chromosomal loop that include the 87A7 *hsp70* locus [13]. Su(Hw) and dCTCF colocalize at several insulator bodies of diploid nuclei, but not in polytene chromosomes, with the Centrosomal Protein 190 (CP190) which is necessary for both insulator body formation and enhancer-blocking activity [14], [15]. BEAF-32 has also been shown to recruit CP190 to specific DNA sites [16], suggesting that loop formation mediated by CP190 might be a common mechanism for insulator function in *drosophila*.

A DNA element displaying features common to other chromatin insulators has been found at the 3′ end of the sea urchin *P. lividus H2A* gene, within the tandem repeat of the early histone unit. As reported, the 462 bp *sns5* fragment is required for regulation of histone gene expression in the early embryo as well as for *H2A* silencing at gastrula stage [17], [18]. A physically separable *sns* fragment of 265 bp, displaying directional enhancer-blocking function in both sea urchin and mammalian cells [19]–[21], was previously identified in *sns5*. Most importantly, *sns5*, but not the enhancer-blocker *sns*, placed in flanking location of a γ-retrovirus vector prevents position effect variegation, improves transgene expression at randomly integration sites in erythroid cells, and by binding erythroid and ubiquitous transcription factors modifies nucleosomal histones to maintain a euchromatic state at the provirus locus [22]. Four protein binding sites have been identified by DNaseI footprinting in the *sns*5 element, namely -*A*, -*B*, -*CT*, and -*D box*, all required for the enhancer-blocking and silencing functions, and none of them resemble the CTCF binding-site consensus sequence [23]. Also the *ArsI* element, the only other insulator so far characterized in sea urchins, does not belong to the CTCF type [24]. It follows that the identification of sea urchin IBPs is of some importance to unravel the mechanism of action of insulators in chromosome organization and gene expression in this species.

There is at least an additional reason to identify *sns5* IBPs, that is, the mechanism of function of *sns5* can be studied within the natural histone gene context. We have in fact presented compelling evidence that its role is to attenuate the *H2A* enhancer in the interaction with the downstream *H1* promoter in order to assure the different level of accumulation of nucleosomal and linker transcripts during sea urchin embryogenesis [25].

In this paper, we describe the identification and functional characterization of a novel homeodomain-containing IBP encoded by the *Compass/Compass-like* locus that is exclusive to the ambulacrarians.

## Results

### Identification and sequence characterization of the COMPASS-like protein family

**Figure 1:**
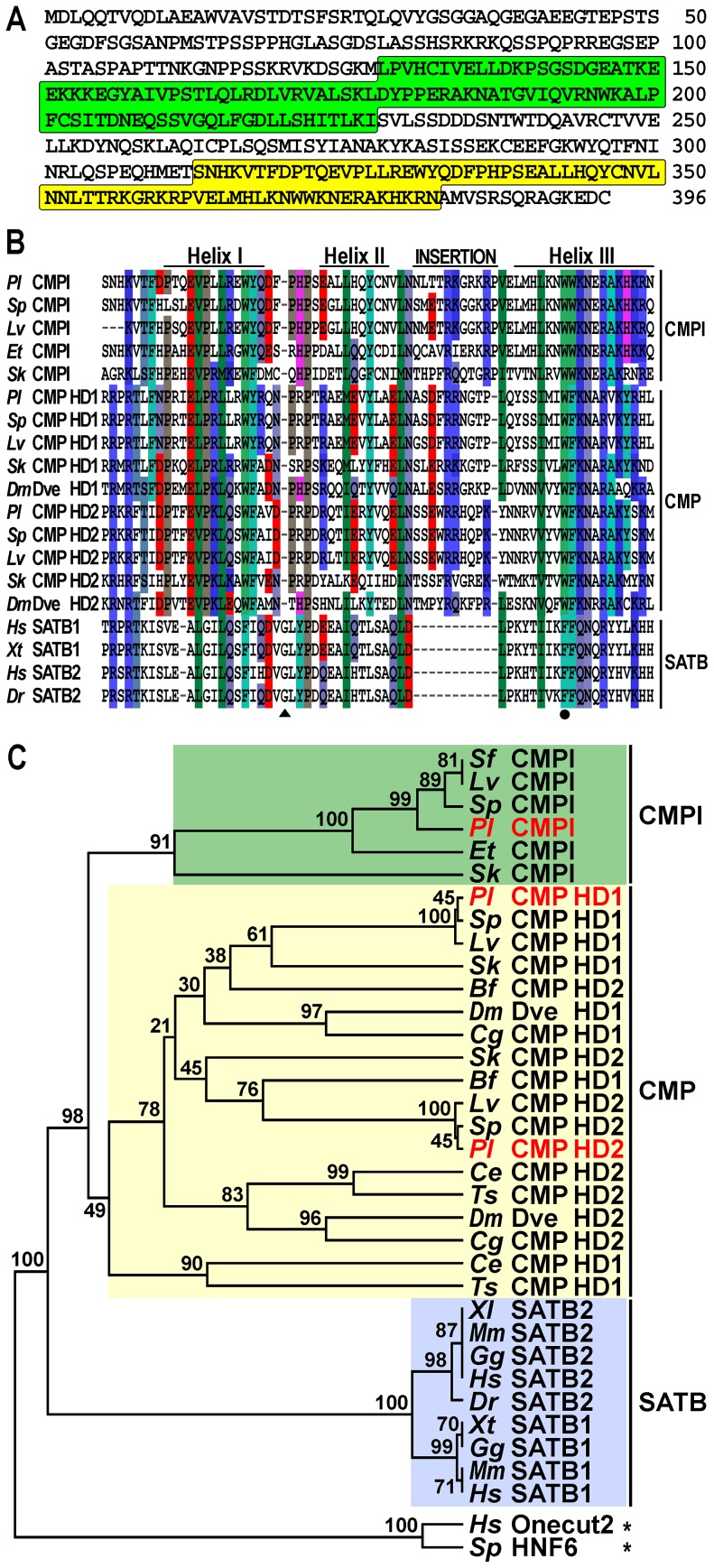
The CMPl protein family. (A) Predicted amino-acid sequence of the *P. lividus* CMPl, with Compass- and Homeo- domain highlighted in green and yellow, respectively. (B) Comparison of the CMPl, CMP and SATB Homeodomains among various species. Differently coloured boxes highlight similarities; dashes represent the gaps inserted for maximal alignment; position 48 is marked by a filled circle, whereas the glycine insertion in SATB sequences is pointed by a triangle. (C) Rooted neighbor-joining tree constructed from the Homeodomains of representative CMPl, CMP and SATB family members. *P. lividus* sequence names are in red; numbers above nodes record percent bootstrap values, while asterisks indicate outgroups. Complete taxonomic names and accession numbers of all the sequences used to elaborate the tree are listed in Supplementary Table S1.

To identify the *trans*-acting factors that interact with the *sns5* insulator in *P. lividus*, we used a yeast one-hybrid genetic assay [26]. Briefly, a cDNA library of N-terminal fusions to the GAL4 activation domain was screened using as bait a yeast strain bearing a stably integrated pentamer of the *boxB cis*-element upstream of both the *HIS3* and *lacZ* reporter genes. From this screening we isolated a ∼2.2 kb cDNA clone encoding a predicted protein of 396 amino acids, which contains a Compass domain followed by an atypical Homeodomain at the C-terminus (Figure 1A). The former domain is shared exclusively among members of the SATB and COMPASS (CMP) protein families [27], [28]. SATB proteins possess an atypical Homeodomain with phenylalanine, instead of tryptophan, at the 48^th^ residue and a single glycine insertion between the first and second helices, whereas CMP proteins contain two atypical Homeodomains with a ten amino-acid insertion between the second and third helices (Figure 1B) [28]. Differently from the above described proteins, the sea urchin predicted protein exhibits a unique atypical Homeodomain bearing an eleven amino acid long insertion between helices II and III (Figure 1B). For these reasons, we have named this newly identified factor COMPASS-like (CMPl).

By blasting the public databases with the sequence coding for the Homeodomain of the *P. lividus* CMPl protein, we show that the above mentioned differences are completely conserved in orthologs of various sea urchin species and in the hemichordate *Saccoglossus kowalevskii* (Figure 1B). Such a high degree of conservation suggests that these proteins play important role(s) in echinoderms and hemichordates, altogether forming the Ambulacraria group of deuterostome metazoans [29].

To clarify the phylogenetic relationship between SATB, CMP and CMPl, we built a neighbor-joining tree using set of Homeodomain sequences from various metazoans. As expected, in this analysis orthologs of the SATB family, which have only been identified in vertebrates [30], [31], comprised a monophyletic clade (Figure 1C). Orthologs of the CMP family, which instead have been described only in invertebrates [31], also formed a clade. Importantly, the ambulacrarian CMPl sequences formed a distinct clade supported by a high bootstrap value, suggesting that they constitute a novel family of proteins. In spite of extensive searches in the currently available databases of several metazoans, additional CMPl orthologs were not identified, indicating that the CMPl family probably exists only in ambulacrarians.

### Alternative splicing and differential accumulation of the *Cmp*/*Cmpl* locus transcripts during sea urchin development

**Figure 2:**
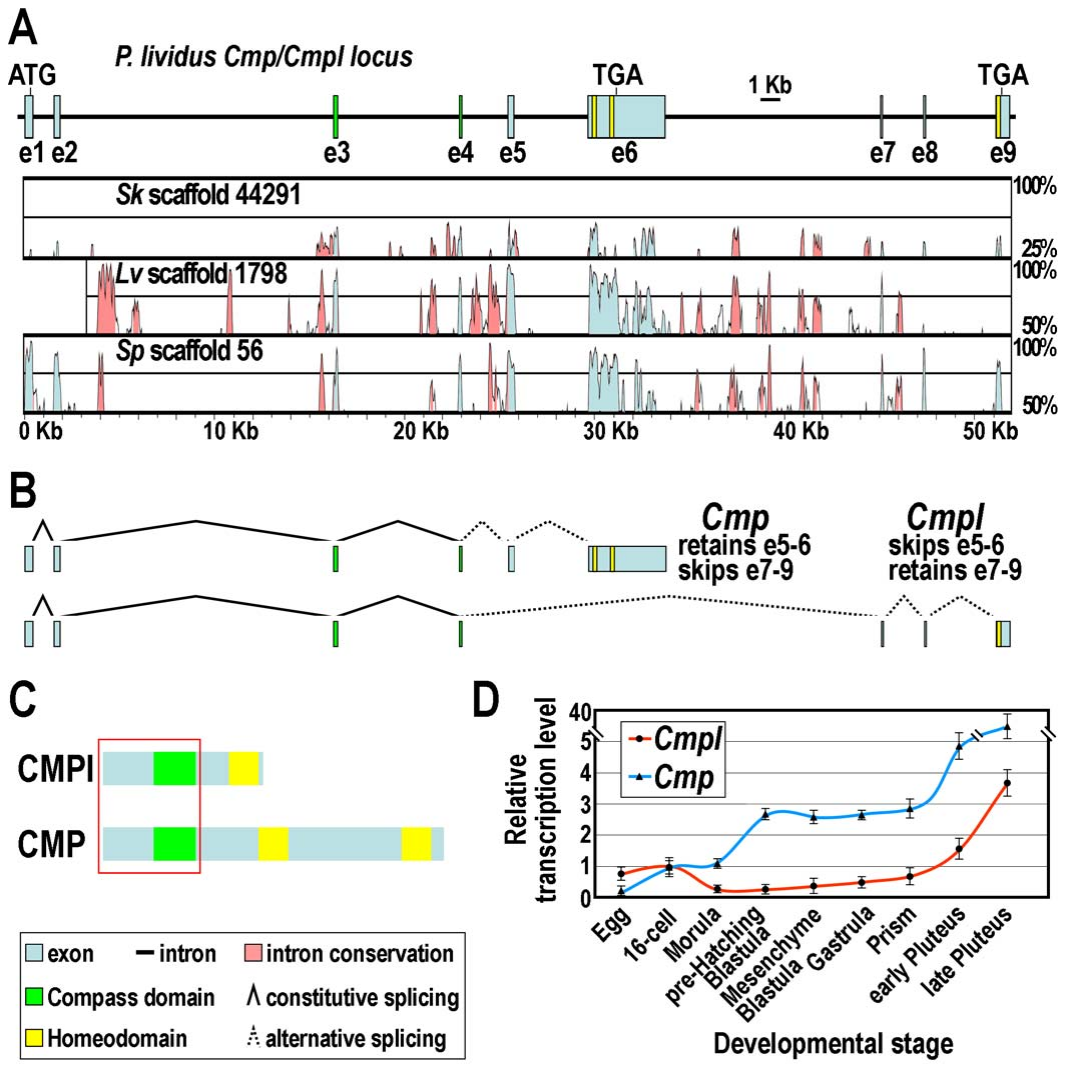
The *Cmp*/*Cmpl* locus and its products. (A) Schematic drawing of the *Cmp*/*Cmpl* gene structure showing the positions of exons. Phylogenetic footprinting analysis is shown beneath the diagram. (B) Structure of the alternatively spliced mRNAs. (C) Diagrammatic representation of the CMPl and CMP protein domain organization. Red square indicates common amino acidic sequence at the N-terminal side. (D) qRT-PCR analysis of *Cmpl* and *Cmp* transcripts throughout *P. lividus* embryogenesis. Values at the various stages are shown as fold difference with respect to the 16-cell stage, which displays roughly equal amount of *Cmpl* and *Cmp* mRNAs. Bars are standard errors for the qPCR replicas.

In order to obtain the nucleotide sequence of the *Cmpl* gene, we BLAST-searched the *P. lividus* genome database (whole genome shotgun assembly v1.0, http://octopus.obs-vlfr.fr/blast/oursin/blast_oursin.php) using the *Cmpl* full cDNA sequence as a query. Several overlapping scaffolds and contigs were isolated (Supplementary Table S1), from which the overall sequence was derived. The gene structure was inferred by aligning the genome sequence with that of the *Cmpl* cDNA and by the use of the Genscan software. We further coupled this analysis to the screening of the available *P. lividus* EST resources. By this approach we retrieved several hits of different size (Supplementary Figure S1). Collectively, these cDNAs harbor a nearly identical 5′-UTR and utilize the same translation initiation sequence, but only one of them almost entirely matched to *Cmpl*. Intriguingly, four of the remaining cDNAs appeared instead larger and highly divergent at the 3′-side compared to the query sequence (Supplementary Figure S1). We noticed that this fragment actually maps within the *Cmpl* gene, being partitioned in a couple of additional exons, namely e5 and e6, which are spliced out in the *Cmpl* mRNA being mutually exclusive with respect to e7–9 (Figure 2A and B).

To assess the conservation of the *Cmpl* locus across ambulacrarians, we extended the BLAST searches to the public genomic databases of the *Strongylocentrotus purpuratus* and *Lytechinus variegatus* sea urchins, and to that of *S. kowalevskii*. From each of the mentioned database, a single genomic scaffold was retrieved (Figure 2A and Supplementary Table S1). Of importance, by phylogenetic footprinting performed by comparison of nucleotide sequences with the VISTA software, we established that the genomic organization of the *P. lividus Cmpl* locus is fully conserved in the two evolutionarily distant sea urchins, as well as in the hemichordate (Figure 2A). Furthermore, the retrieving of a single *Cmpl* hit from the fully completed genome sequence of *S. purpuratus* leads us to presume that *Cmpl* is most probably a single copy gene in sea urchins.

Sequence analysis revealed that, as expected, the protein encoded by the largest splice variant was identical to CMPl for 233 amino acids at the N-terminal side, including the Compass domain, but strongly diverged in the C-terminal region. Most notable is the presence of two atypical Homeodomains, with an insertion of ten amino acids between helices II and III (Figure 2C and Figure 1B). On the bases of these findings, coupled to the phylogenetic analysis based on Homeodomain sequences (Figure 1B and C), we designated this protein as the sea urchin CMP ortholog. Therefore, and unexpectedly, the genetic information for CMPl and CMP proteins partially overlaps in representative genomes of both the ambulacrarian taxa.

We then looked by qPCR at the time-course of accumulation of the two splice forms, utilizing primers that distinguish them. As shown in Figure 2D, both transcripts are maternally stored in the unfertilized egg and present at all stages of development. However, *Cmpl* mRNA is accumulated in the embryo at about a three- to ten-fold lower level than is the *Cmp* mRNA. After fertilization, *Cmpl* transcript abundance declines throughout the very early cleavage (up to morula stage), followed by a slight and steady increase until the prism stage. At this time, a later sharp burst in the message prevalence is detected through the pluteus larva.

The *Cmp* transcript is the most abundant and it is accumulated in the embryo following three main phases of expression. Just after fertilization, the mRNA level rapidly raises to peak at the 16-cell stage. A second increase in transcript level occurs approximately from the morula stage, to peak as the pre-hatching blastula is approached. The terminal phase of mRNA accumulation begins at the prism stage, by which time a dramatic climb in the transcript abundance is observed. Thus, these results clearly established distinct temporal expression patterns for the alternative splice products of the *Cmp*/*Cmpl* locus.

Altogether, our findings indicate that the genomic organization of the *Cmp*/*Cmpl* locus is evolutionary conserved across ambulacrarians, and that the mRNAs generated by the alternative spliced *Cmp*/*Cmpl* transcript exhibit distinct temporal expression profile in the sea urchin embryo.

### CMPl, but not CMP, specifically binds the *boxB cis*-element *in vivo*

**Figure 3:**
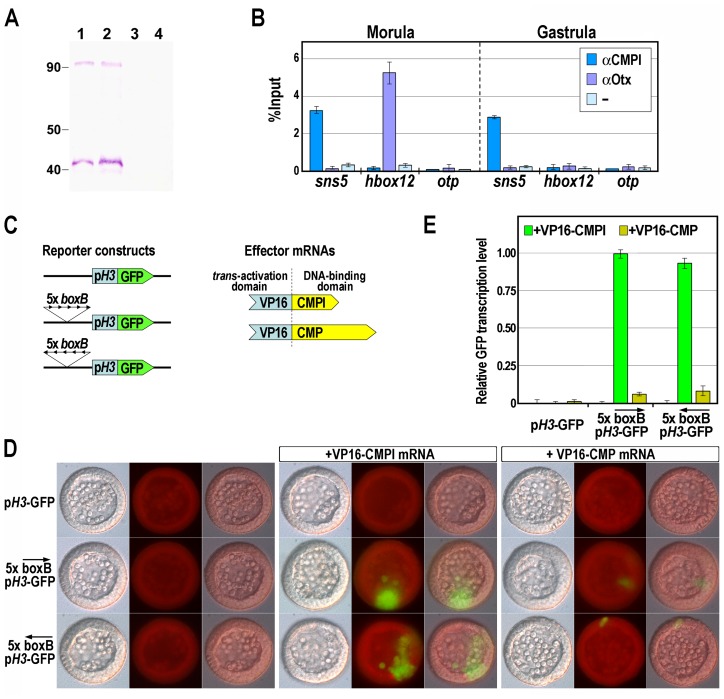
Association of the CMPl protein to the *boxB cis*-element on *sns5*. (A) Western blot analysis to test the specificity of the anti-CMPl antibody. Nuclear extracts from morula (lanes 1, 3) and gastrula (lanes 2, 4) embryos were fractioned by SDS-PAGE, blotted on nitrocellulose membrane and incubated with anti-CMPl antibody (lanes 1, 2) or pre-immune serum (lanes 3, 4). (B) ChIP-qPCR analysis of the *sns5* occupancy by CMPl. ChIP assays were performed on chromatin extracted from embryos at the indicated stages and precipitated with antiserum against CMPl or Otx, or incubated without adding antibodies (−), followed by qPCR amplification of an *sns5* fragment containing the *boxB* sequence, or *hbox12* and *otp* promoter fragments. As described previously, the association of the Otx transcription factor to its binding site within the *hbox12* promoter correlates with the *hbox12* transcriptional state [32]. Data are normalized according to the percent of input method. Bars are as in Figure 2D. (C) Scheme of the reporter and effector constructs used in the *trans*-activation assay. (D) *Trans*-activation analysis in developing sea urchin embryos observed at the mesenchyme blastula stage. DIC, epifluorescence and merged images, respectively ordered from top to bottom, are shown for each embryo. (E) GFP expression levels assessed by qPCR in transgenic embryos at the mesenchyme blastula stage. Graphs show n-fold changes in mRNA expression level of GFP based on the normalized threshold cycle numbers, with respect to the 5×*boxB-pH3*-GFP/VP16-CMPl co-injected embryos. Bars are as in Figure 2D.

To ascertain the binding activity of CMPl to the *sns5* chromatin in sea urchin embryos, we performed quantitative ChIP assays. To this end, we expressed different portions of the CMPl protein in *E. coli*. As the fragment corresponding to the N-terminal 98–270 amino acid residues gave the maximum yield of the protein in a soluble form, we have generated a polyclonal antibody against this peptide. Being the first 134 residues of this peptide shared by CMPl and CMP, we predicted that the anti-CMPl antibody should rather be able to react with both proteins. Indeed, in western blot assay, the antibody recognized two distinct protein bands at roughly 40 and 90 kDa in sea urchin nuclear extracts at morula and gastrula stages (Figure 3A). These molecular weights were congruent with those predicted for CMPl and CMP proteins, respectively, whereas no reaction occurred with the pre-immune serum (Figure 3A).

Chromatin containing the *sns5* region was consistently precipitated by the affinity-purified anti-CMPl antibody, in samples obtained from cultures of embryos at morula and gastrula stages (Figure 3B; see Materials and Methods). As a negative control, we selected two additional genes, *hbox12* [32] and *otp* [33], [34], that do not share significant sequence similarity with *sns5* in their promoters. As expected, both genes were clearly negative to CMPl occupancy from the same ChIP preparations (Figure 3B). Furthermore, only negligible amounts of *sns5* sequences were precipitated from chromatin of both developmental stages by the unrelated antiserum from the same host species against the Otx regulator [32], used as control. Overall, these results point out the specific and constitutive association of CMPl to *sns5* sequence in the native chromatin. However, as the antibody effectively recognizes epitopes common to both CMPl and CMP, these experiments may represent the full impact of both proteins on the *sns5* chromatin.

We addressed this question by performing a *in vivo trans*-activation assay. To this purpose, pentamer of the *boxB cis*-element was introduced, in both orientations, upstream of the *H3* minimal promoter in the *pH3*-GFP vector, to obtain the 5×*boxB*-*pH3*-GFP reporter constructs (Figure 3C). As effectors we used synthetic mRNAs encoding for a forced transcription activator, in which the activation domain of the viral VP16 protein was joined to the DNA binding domain of either CMPl or CMP. Each transgene, alone or in combination with a chimeric effector mRNA, was then microinjected into sea urchin zygotes, embryos were allowed to develop and scored for GFP expression. As shown in Figure 3D, the control *pH3*-GFP vector and the 5×*boxB*-*pH3*-GFP constructs were not expressed in the absence of effectors in all of the injected embryos (n>300). As expected, and in agreement with the one-hybrid and ChIP results, a significant fraction of embryos (52%, n>300) injected with the reporter construct along with VP16-CMPl mRNA exhibited patches of clonal GFP-expression, irrespectively of the orientation of the *cis*-acting element on the transgene. By contrast, expression of the reporter was barely detectable in a minor fraction of embryos (5%, n>300) co-injected with equal amounts of the VP16-CMP mRNA (Figure 3D). qPCR measurements further confirmed that GFP expression was weakly evoked in these specimen, with respect to the VP16-CMPl co-injected embryos (Figure 3E). Altogether, these results support the contention that most, if not all, of the *boxB* binding sequences specifically recruits CMPl *in vivo*.

**Figure 4:**
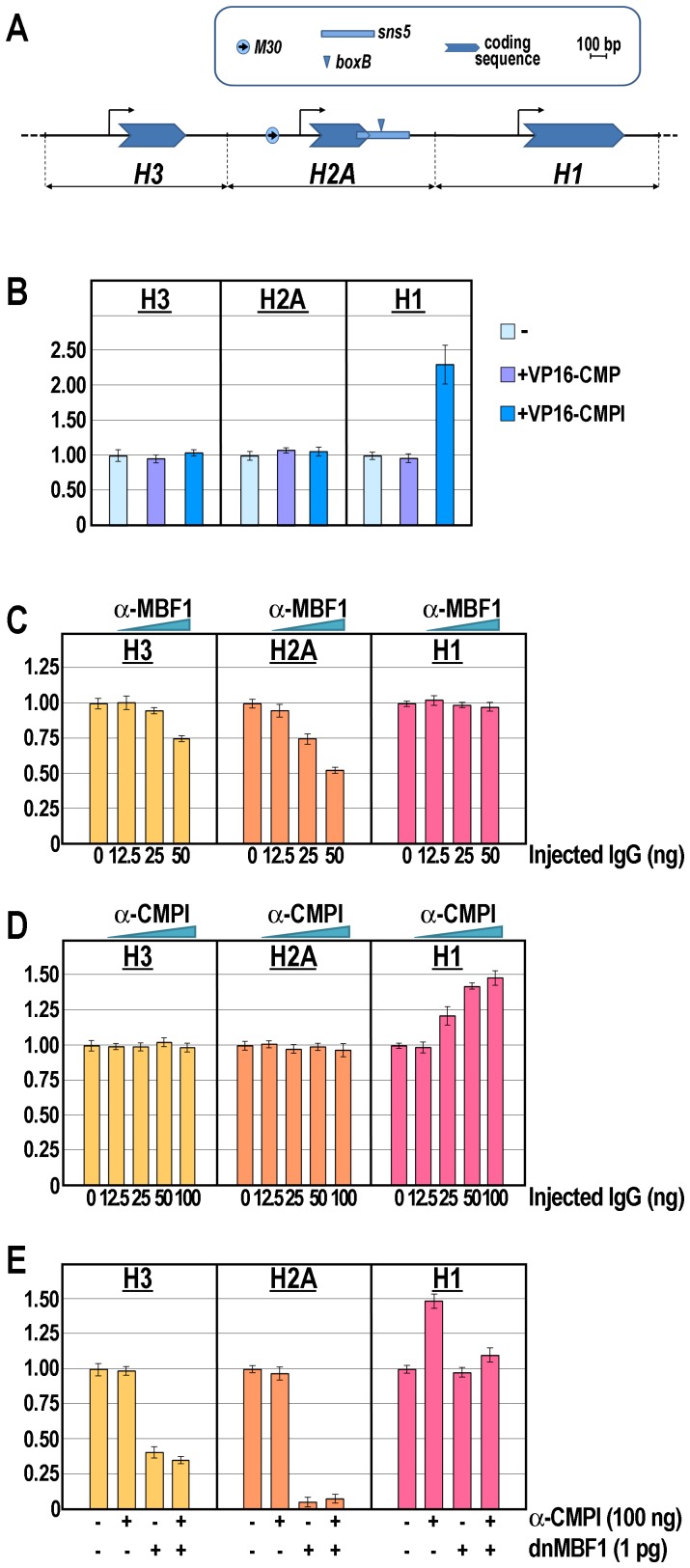
Knock-down of the *M30*-enhancer and/or *sns5*-insulator functions and effect on the endogenous early histone gene transcription. (A) Annotated map of the *P. lividus* early *H3*, *H2A* and *H1* histone genes, highlighting the *cis*-regulatory sequence elements. The horizontal black line represents the genomic DNA, while the bent arrows denote the putative transcription start site. (B–E) qPCR analysis of histone gene transcription carried out in embryos at morula stage injected with the VP16-CMPl/VP16-CMP transcripts (B), or with increasing amounts of the α-MBF1 (C) or α-CMPl (D) antibodies, or the chimeric dnMBF1 mRNA together with saturating dosage of the α-CMPl antibody (E). Graphs show n-fold changes in mRNA expression level of histone genes based on the normalized threshold cycle number of injected embryos compared to that of the uninjected control embryos. Data were derived from two independent microinjection experiments and each bar represents the average of triplicate samples from the two batches of embryos.

Further insights were obtained by examining the specificity of binding of CMPl versus CMP to the *boxB* element in the natural chromatin context of *sns5*, within the histone gene cluster. The DNA replication-dependent sea urchin early histone genes are organized in a single large cluster made up of almost 2000 tandem repeats of the 5′-*H2B-H3-H2A-H1-H4*-3′ unit [35]. Coordinate transcription of these genes is limited to the cleavage and reaches its maximum at the morula stage. The *M30 cis*-regulatory sequence, upstream the *H2A* promoter, upon binding of the MBF1 activator displays a bidirectional enhancer activity [25], [36]. Remarkably, as we previously shown [25], the *H1* promoter is shielded by the *M30* enhancer activity by the *sns5* insulator, which is located at the 3′end of the *H2A* transcription unit (Figure 4A).

Sea urchin zygotes were microinjected with either the VP16-CMPl or the control VP16-CMP synthetic transcripts. Then, the expression of the *H3*, *H2A* and *H1* genes was analyzed by qPCR at the morula stage. As expected, the injection of the VP16-CMP transcript had no detectable effect on histone genes expression (Figure 4B). Likewise, the mRNA levels of *H3* and *H2A* did not show relevant change following the injection of the VP16-CMPl transcript. By contrast, the number of molecules of *H1* mRNA was more than double in embryos expressing VP16-CMPl (Figure 4B). The most obvious explanation for the enhancement of *H1* expression is that VP16-CMPl acted as a transcriptional activator on the *H1* promoter. Alternatively, VP16-CMPl, by competing with the binding of the endogenous CMPl protein, impaired the enhancer-blocking activity of the *sns5* element, thus allowing the *H2A* enhancer to act on the *H1* promoter. This hypothesis is in line with our previous *in vivo* competition assays showing that inhibition of the *sns5* led to up-regulation of only the *H1* gene [25]. In conclusion, whatever is the mechanism, these experiments, as well as ChIP and *trans*-activation assays, strongly suggest that CMPl, but not CMP, associates to the *boxB* site *in vivo*.

### CMPl binding to the *boxB* site mediates the *sns5* enhancer-blocking activity

The finding that CMPl occupies the *sns5* chromatin provided an opportunity to examine the function of an IBP in its natural gene context. To challenge CMPl activity, increasing amounts of the affinity-purified anti-CMPl antibodies, or control antibodies against the *H2A* enhancer binding factor MBF1, were injected into the sea urchin zygotes and histone gene expression analyzed by qPCR at the morula stage. In these experiments, injection of anti-MBF1 provoked a dose-dependent negative effect on the expression of the nucleosomal *H2A* and *H3*, but not the linker *H1*, genes (Figure 4C), excluding a unspecific effect of the injection. Also, it should be noted that *H2A* was more strongly affected compared to *H3*. These results are in agreement with those previously obtained by the *in vivo* inhibition of the *H2A* enhancer [25].

In strict accordance with previous findings [25 and this paper], both *H2A* and *H3* mRNAs did not vary their abundance upon injection of different doses of anti-CMPl (Figure 4D), while the number of *H1* mRNA molecules increased proportionally with the augmentation of the CMPl antiserum (Figure 4D). Taken together, these findings strongly point a role of the CMPl factor in mediating the *sns5* insulator function by binding to the *boxB cis*-element.

Furthermore, we predicted that the block of the *H2A* enhancer function might counteract the rise of *H1* expression due to inhibition of CMPl binding to *sns5*. Indeed, the results showed in Figure 4E fulfilled this assumption, as co-injection of dnMBF1 mRNA, the dominant-negative version of MBF1 [25], along with saturating amounts of anti-CMPl caused a significant drop in the prevalence of *H1* transcripts. Remarkably, in these embryos the number of mRNA molecules for the linker histone was comparable to that of the control uninjected embryos, confirming the independence of *H1* transcription from the MBF1/enhancer positive input. Once again, the mRNA abundance of *H2A* and *H3* (although to a lesser extent than *H2A*) decreased with the microinjection of dnMBF1, irrespectively of the anti-CMPl presence (Figure 4E).

On the basis of these results, we conclude that recruiting of the CMPl factor to the *boxB cis*-element is critical for the enhancer-blocking activity of the *sns5* insulator during the early embryogenesis of the sea urchin.

## Discussion

Chromatin insulator functions rely upon the assembly of protein complexes initiated by insulator DNA-binding proteins (IBPs). Although few IBPs have been identified so far, recognition motifs for IBPs represent one of the most conserved non-coding DNA elements in metazoan genomes [37], [38], indicating that these proteins have a critical role in transcriptional regulation. The functions of the vast majority of known IBPs apparently converge as chromatin organizer into that of the paradigmatic CTCF protein. Nevertheless, CTCF orthologs are apparently absent in organisms such as yeast and plants [39], which do have insulator elements embedded in their genome. As mentioned, two insulators have been characterized in sea urchins, namely *sns5* and *ArsI*, and none of them do contain a CTCF binding consensus sequence, raising the question of whether or not a sea urchin CTCF ortholog is involved in enhancer-blocking and chromatin organization as it does in *Drosophila* and vertebrates. Of importance, it should be emphasized that a DNA sequence erroneously annotated in the *S. purpuratus* database as a sea urchin CTCF-related gene rather encodes the well characterized Zinc-finger MBF1 transcription activator [36]. Hence, it remains as a possibility that the CTCF ortholog has been lost during evolution in some echinoderms, as occurred in several nematodes [39].

### CMPl is a compass- and homeo-domain containing regulator of a novel family

Previous studies in the sea urchin model led to the identification of several candidate proteins (such as ISWI, Sin3A, PARP1, and more) that are recruited on the *ArsI* insulator [40]. Although all these factors are probably part of protein complexes required for enhancer-blocking activity of *ArsI*, none of them directly binds the insulator DNA sequence and therefore should not be considered a sensu stricto IBP. Here we have described the identification and functional characterization of CMPl which, to our knowledge, represents the first IBP of a non-model organism. Our molecular analyses indicate that although *Cmpl* is a single copy gene in the sea urchins, at least two distinct transcripts exist, due to alternative RNA splicing. They encode the CMPl and CMP proteins that are identical in their N-terminal sequences, where the Compass domain is located. Such a domain is followed by one or two atypical Homeodomains, respectively in CMPl and CMP. The sea urchin CMP and CMPl proteins belong to distinct families. Indeed, the former, and likely the most abundant, protein displays features of the COMPASS family, containing two ^48^W-type Homeodomains, each embedding a decapeptide insertion between helices II and III. Although displaying a ^48^W signature, the single Homeodomain of CMPl exhibits instead an unusual K/R-rich insertion of eleven residues which is not found in any other known Homeodomain protein but is conserved in sea urchins and *S. kowalevskii*. As for most of the *Drosophila* IBPs, apparently, there are no sequence homologs of CMPl in vertebrates. Thus CMPls constitute an additional, previously not described, ambulacrarian-specific family of proteins within those containing both the Compass- and Homeo-domain.

### *Cmp*/*Cmpl* locus and the monophyly of the Ambulacraria

Although the CMP protein is rather conserved among invertebrates, our phylogenetic analysis strongly suggests that hemichordates and echinoderms share a unique genomic organization at the *Cmp*/*Cmpl* locus. Consequently, the CMPl protein is found neither in chordates nor in protostomes. The monophyly of Ambulacraria is supported by three morphological characters: a trimeric arrangement of the adult coeloms, an axial complex with hydropore, and a dipleurula larva with neotroch [41]. At the molecular level, monophyly is also supported by 18S rDNA gene analyses [41]–[44], a unique mitochondrial gene code [45]–[46], the presence of three distinct posterior Hox genes [47], and now the possession of the *Cmp/Cmpl* locus.

The functional significance of the emergence and loss of CMPl, respectively in ambulacrarians and chordates, is not clear at the moment. On the other hand, the *Cmp* gene is thought to have a highly flexible behavior during evolution. According to a current model, *Cmp* genes had emerged from a common ancestor with the POU class homeobox genes, acquiring the insertions in the COMPASS- and Homeo-domain [31]. In the lineage to vertebrates, *SATB* genes emerged and the genomic structure changed after the divergence of the amphioxus and vertebrate ancestors. *SATB* gene may have arisen from the *Cmp* gene by domain shuffling between *Cmp* and *Onecut* genes and eventually the *Cmp* gene was lost from the ancestral vertebrate [31]. In light of this scenario, the genomic configuration of the ambulacrarian *Cmp*/*Cmpl* locus could be emerged by similar mechanisms.

Last, but not least, many insulator proteins are known to have multiple functions, and a wider functional analysis of the possible CMPl functional contribution to the sea urchin embryogenesis, outside the *sns5* region, should help in the elucidation of the matter. In any case, this study provides an additional step towards an understanding of the evolution of *Cmp*, *Cmpl* and *SATB* genes in lower deuterostomes.

### *sns5*-directed binding activity and functional role of CMPl

The specific binding of CMPl to the *boxB cis*-element of the *sns5* chromatin insulator is substantiated by several lines of evidence. First, a cDNA encoding the CMPl protein was recovered following a yeast one-hybrid assay, using the *boxB* sequence as bait. Second, constitutive occupancy of the *sns5* chromatin by the CMPl protein was demonstrated by ChIP assay, using a CMPl antiserum. Third, specific interaction between the CMPl Homeodomain and the *boxB* element was further verified by a *trans*-activation assay in which *boxB* was placed upstream of the GFP reporter and the resulting transgene injected together with an mRNA encoding the CMPl Homeodomain fused to VP16. Fourth and most important, the expression of the VP16-CMPl chimeric protein, by competing with the endogenous CMPl, acts as either a *trans*-activator of the *H1* promoter or as inhibitor of the enhancer-blocking activity of *sns5*, or both. Whatever is the mechanism, such a result definitively proved the specific association of the CMPl Homeodomain with the *sns5* native chromatin.

Intriguingly, this result clearly indicates that the Homeodomain alone is sufficient for CMPl effective DNA binding, in apparent contrast to the Compass domain-mediated oligomerization required for SATB1 to bind specific DNA sequences [48]. Although the Compass domain has been initially assimilated to a PDZ-like domain, a recent structural study indicated that it rather resembles a classic ubiquitin domain, even in the absence of sequence homology [49]. According to this study, the Compass domain mediates homo-tetramerization of SATB1 in solution. In particular, two dimers are joined together through multiple hydrogen bonds and hydrophobic interactions within their interfaces. Among these reciprocal interactions, the ^97^E^98^F^162^H residues are necessary for the formation of a hemi-dimer, and the ^136^K^137^W^138^N triad is important for the formation of the other dimer.

A sequence alignment of Compass domain across species shows that these residues are all highly conserved among SATB proteins (Supplementary Figure S2), indicating that the Compass domain may have similar biological functions in vertebrates. By contrast, residues at positions 97, 98, and 162 are divergent in ambulacrarian sequences, and the ^136^K^137^W^138^N tripeptide exhibits an inverted orientation in the primary sequence (Supplementary Figure S2). The observed changes in key residues of the Compass domain could explain the different behaviour in DNA binding activity between CMPl and SATB1.

Expression of a CMP Homoeodomain-VP16 chimera in the developing embryo only modestly affected GFP expression in the *trans*-activation assay, and failed to prejudice *sns5* enhancer-blocking function. Altogether, these findings lead us to conclude that no, or maybe very weak, interaction occurs between CMP and the *boxB cis*-element. It is known that the transcription factor LFB1/HNF1 contains a twenty-one amino-acids insertion between helices II and III which forms a large extra-loop that does not affect the overall structure of the Homeodomain [50]. On this basis, we speculate that the different-size insertions in CMPl and CMP Homeodomains do not participate in DNA interactions. Rather, we reckon that substitution of specific amino acids in Homeodomain sequences may account for differences in DNA binding affinity. Indeed, the Homeodomain of CMPl has a certain sequence similarity with those of CMP (58% and 66%, respectively; Figure 1B) but, notably, significant differences are detectable even in the helix III, which is known to be responsible for the discrimination of binding sequences [51]–[52].

The identification of CMPl in the sea urchin is of some importance to unravel the mechanism of action of insulators in chromosome organization and gene expression in this species. A principal criticism on studying insulators is that, often, the assays are performed by using artificial constructs in a chromosome context different than that of theirs, and it is known that the enhancer/promoter combination influences the activity [53]. In sharp contrast, our experiments provide a clear demonstration of the CMPl-dependent anti-enhancer function of the *sns5* insulator in its natural chromatin context. Indeed, we have shown that the inhibition of the CMPl/*boxB* interaction, either by expressing the VP16-CMPl chimera or by titrating the CMPl factor through injection of specific antibodies, allowed an up-regulation of only the endogenous *H1* gene. Most importantly, impairment of the *H2A* enhancer activity by expressing the forced repressor dnMBF1 restores the normal level of *H1* transcripts in embryos in which CMPl is titrated by saturating amounts of the specific antiserum.

Altogether, the results presented in this article strongly suggest that the recruitment of the CMPl protein on the *sns5* insulator is absolutely required for buffering the *H1* promoter from the *H2A* enhancer activity, and this, in turn, accounts for the different level of accumulation of early nucleosomal and linker transcripts.

We have previously published several manuscripts analyzing the properties of the *sns5* insulator in mammalian cells [20]–[22]. The finding that CMPl is an ambulacrarian-specific factor raises the paradoxical question of how *sns5* works as an insulator in cells in which CMPl is absent. For completeness, it should be also remarked that neither the *sns5* sequence does exist in the normal vertebrate genome but, if introduced by means of specific vectors, it is bound by several nuclear factors that are normally not recruited in sea urchin cells [21], [22]. In particular, through EMSA and ChIP assays we have demonstrated the occupancy of the *boxB* element by the Oct1 Homeodomain-containing regulator [21], [22]. In light of this evidence, an explanation to the above mentioned paradox could be that the unconventional recruitment of Oct1 on *sns5* in mammalian cells somehow compensates for the lack of CMPl. A pertinent study in transgenic plants documents the enhancer-blocking activity of BEAD-1, a CTCF-dependent human-derived insulator [54], [55]. As reported above, no functional equivalents of CTCF factor and correspondent binding sites have been identified in plants. However, a large number of zinc-finger factors exhibit at least some degree of similarity at the amino acid level with the zinc-fingers of the vertebrate CTCF proteins [56] and may provide a similar function. In any case, the fact that insulators retain their activity in a unnatural host organism implies that at least a proportion of the insulator machinery in eukaryotes may be evolutionarily conserved.

## Materials and Methods

### Yeast one-hybrid screening

poly(A)^+^ RNA was extracted from total RNA of *P. lividus* embryos at the gastrula stage and cDNA library was made by using the Matchmaker One-Hybrid System kit (Clontech). Briefly, poly(A)^+^ RNA was reverse transcribed and the cDNA products were inserted into a shuttle vector, pGAD10, containing the GAL4 activation domain. Transformation of *E. coli* DH5α cells yielded >6×10^5^ independent colonies. Pentamer of the 38 bp *boxB* sequence was used as the bait to select DNA-binding domains encoded in the library. The bait was inserted into pHISi-1 and pLacZi reporter vectors, and the recombinant plasmids were introduced sequentially into the genome of the yeast strain YM4271. Transformants were tested for growth on medium lacking histidine (SD/−His) in the presence of increasing concentrations of 3-aminotriazol (3-AT). Cells whose growth was inhibited by 5 mM 3-AT were selected as the host for the library screen. Transformation with the library was carried out by using LiCl-PEG, and transformants grown on SD/−His and SD/−Leu selective medium were tested for β-galactosidase activity. Plasmids from positive yeast clones were isolated by homogenization with glass beads and then individually transferred into DH5α cells for amplification. To eliminate false positives, these plasmids were separately introduced into yeast cells containing either the *boxB* bait or the p53 binding site, and transformants were tested for β-galactosidase activity. In this assay, a single plasmid conferred expression only in the *boxB* host. Sequence analysis revealed that such a plasmid harbored a ∼2.2 kb cDNA insert encoding for CMPl (Genbank accession number: KF421245).

### Sequence analysis

BLASTP, BLASTX, and TBLASTN searches in public sequence databases (http://blast.hgsc.bcm.tmc.edu/blast.hgsc; http://www.molgen.mpg.de/~ag_seaurchin) with *P. lividus* CMPl and CMP as queries were performed and yielded candidates for many phyla. In some cases, a CMP coding region was deduced by Genscan analysis of genomic contigs. Protein domain architecture was defined via use of the Pfam (http://pfam.sanger.ac.uk) and SMART (http://smart.embl-heidelberg.de) databases. Multiple sequence alignments were generated using ClustalW, the output file was formatted using BioEdit, and neighbor-joining tree was constructed using the MEGA software package (http://www.megasoftware.net). The reliability of branching points was inferred by bootstrapping using 1000 replicates. Genomic sequence comparisons for phylogenetic footprinting were performed with the VISTA platform (http://genome.lbl.gov/vista/index.shtml), using window size varied between 50 and 100 bp, with 70% and 50% conservation respectively for sea urchins and *S. kowalevskii*.

### Antibody production, ChIP and qPCR

A DNA fragment corresponding to amino acids 98–270 of CMPl was subcloned into the pGEX-4T-1 expression vector to create a glutathione-S-transferase (GST) fusion protein. This protein was affinity purified from bacterial extracts and then used to generate a rabbit polyclonal antibody (Eurogentec, BE). After serum preincubation with a sepharose-4B column bound to the GST protein, the anti-CMPl antibody was purified by sepharose-4B affinity columns bound to the GST-CMPl protein. The specificity of the antibody was assayed by western blot detection against GST-CMPl and Thioredoxin-CMPl proteins.

ChIP experiments were performed essentially as described in [32], with minor modifications. Briefly, formaldehyde cross-linked sea urchin embryos at morula and gastrula stages were incubated in cell lysis buffer (10 mM HEPES pH 8.0 and 85 mM KCl, containing the following protease inhibitors: 0.5% NP40, 1 µg/ml leupeptin, 1 µg/ml aprotinin, 1 mM PMSF), for 10 min on ice. Nuclei were then resuspended in nuclear lysis buffer (50 mM Tris-HCl pH 8.1, 10 mM EDTA and 1% SDS, containing the same protease inhibitors as in cell lysis buffer) and incubated on ice for 10 min. Chromatin was sonicated using a Bandelin Sonopuls ultrasonic homogenizer to an average fragment size of 150 to 500 bp, as determined by agarose gel electrophoresis. To reduce non-specific background, the samples were diluted into five volumes of ChIP dilution buffer (16.7 mM Tris-HCl pH 8.1, 167 mM NaCl, 0.01% SDS, 1.1% Triton X-100, 1.2 mM EDTA, plus proteinase inhibitors) and incubated with 100 µl of a salmon sperm DNA/protein A-sepharose slurry for 1 h at 4°C, with mixing. Ten percent of chromatin was withdrawn (Input) and processed as the immunoprecipitated chromatin. Aliquots of chromatin containing 25 µg of DNA were incubated in the absence of antibodies (as a negative control) or either with the anti-CMPl or the anti-Otx serum overnight at 4°C. The immune complexes were adsorbed to protein A-sepharose beads, which were sequentially washed with a low salt buffer (0.1% SDS, 1% Triton X-100, 2 mM EDTA, 20 mM Tris-HCl pH 8.1, 150 mM NaCl), a high salt buffer (0.1% SDS, 1% Triton X-100, 2 mM EDTA, 20 mM Tris-HCl pH 8.1, 500 mM NaCl), a LiCl buffer (0.25 M LiCl, 1% NP40, 1% deoxycholate, 1 mM EDTA, 10 mM Tris-HCl pH 8.0) and twice in 1×TE buffer (10 mM Tris-HCl, 0.1 mM EDTA pH 8.0). The immune complexes were eluted with the elution buffer (1% SDS, 0.1 M NaHCO_3_), digested with RNase at 37°C and treated with proteinase K in 0.3 M NaCl at 65°C for 4 h to reverse the cross-links. DNA from chromatin samples was extracted with phenol/chloroform, precipitated with ethanol and dissolved in 50 µl of water. DNA samples were then quantified by readings in a Qubit Fluorometer (Invitrogen) using the Quant-iT dsDNA HS assay kit (Invitrogen).

The enrichment of histone regulatory sequences in 100 pg aliquots of genomic DNA purified from the precipitated chromatin fractions was examined by qPCR as described previously [25], [32]. qPCR experiments were performed from two different batches and all reactions were run in triplicate on the 7300 Real-Time PCR system (Applied Biosystems) using SYBR Green detection chemistry. ROX was used as a measure of background fluorescence and, at the end of the amplification reactions, a ‘melting-curve analysis’ was run to confirm the homogeneity of all amplicons. Calculations from qPCR raw data were performed by the RQ Study software version 1.2.3 (Applied Biosystems), using the comparative Ct method. Primer efficiencies (i.e., the amplification factors for each cycle) were found to exceed 1.9. In every experiment, a no-template control was included for each primers set. Primers used in this study are listed in Supplementary Table S2.

The amounts of *Cmp*/*Cmpl* and histone gene transcription in control and injected embryos were evaluated as follows. Total RNA from batches of 150 embryos at the desired stage was extracted by using the Power SYBR Green Cells-to-C_T_ kit (Ambion) and reverse transcribed following the manufacturer's recommendations. The resulting cDNA sample was further diluted and the equivalent amount corresponding to one embryo was used as template for qPCR analysis, using the primers indicated in Supplementary Table S2. A *cytochrome oxidase* or the *mbf1* mRNA, which are known to be expressed at a constant level during development [25], [32], were used to normalize all data, in order to account for fluctuations among different preparations.

### DNA constructs, synthetic transcripts and microinjection

The *pH3*-GFP DNA plasmid was constructed as follows. A PCR fragment harboring the *H3* minimal promoter (spanning from −62 to +60 with respect to the transcription start site) was inserted in the plasmid polylinker of a pGL3 vector containing the GFP coding sequence. The 5×*boxB*-*pH3*-GFP reporter constructs were obtained by shotgun cloning of ligated double-stranded oligonucleotides, bearing the *boxB cis*-regulatory sequence, upstream of the *H3* promoter of the *pH3*-GFP plasmid. The VP16-CMPl and VP16-CMP effector constructs were obtained by fusing the DNA-binding domain coding sequences of either CMPl or CMP to those of the VP16 activation domain cloned in the CS2+nls expression vector. All DNA clones were checked by sequencing.

Capped mRNAs were synthesized from the linearized pCS2-constructs using the mMessage-mMachine kit (Ambion). Purified RNAs were resuspended at 0.5 mg/ml and 2 pl were then microinjected either alone or in combination with the linearized *pH3*-GFP and 5×*boxB*-*pH3*-GFP constructs.

Microinjection was conducted as previously described [57]–[58]. In the *trans*-activation assay, more than 100 injected embryos per experiment were scored for GFP expression at the mesenchyme blastula stage and each experiment was repeated three times with different batches of eggs. As a control of the expression of the injected transgenes, we used an actively transcribed GFP construct driven by the full promoter of the *hbox12* gene [32]. Images were captured with a Leica DC300F digital camera. As for *in vivo* titration assays with antibodies, affinity purified rabbit polyclonal IgG reacting with either CMPl or MBF1 were diluted to a final concentration of 12.5–100 ng/pl in ultrapure water containing 30% glycerol, and eventually injected into sea urchin zygotes.

## Supporting Information

Figure S1 Nucleotide sequence multiple alignment of the *Cmpl* full cDNA and several ESTs retrieved from the *P. lividus* database. Yellow square indicates the translation start codon.
(TIF)

Figure S2 Multiple comparison of the Compass domain of representative SATB, CMP and CMPl family proteins. The position of the residues involved in SATB1 oligomerization are indicated above sequences. Complete taxonomic names and accession numbers of all the sequences used in the alignment are listed in Supplementary Table S1.
(TIF)

Table S1 Complete taxonomic names and accession numbers of the sequences used in this study.
(DOC)

Table S2 List of gene-specific oligonucleotides used in the qPCR analyses.
(DOC)
