# Supplementary figures and images for: The Compass-like Locus, Exclusive to the Ambulacrarians, Encodes a Chromatin Insulator Binding Protein in the Sea Urchin Embryo
Source: PLoS Genet. 2013 Sep 26;9(9):e1003847. doi: 10.1371/journal.pgen.1003847 (PMC3784565; doi:10.1371/journal.pgen.1003847)

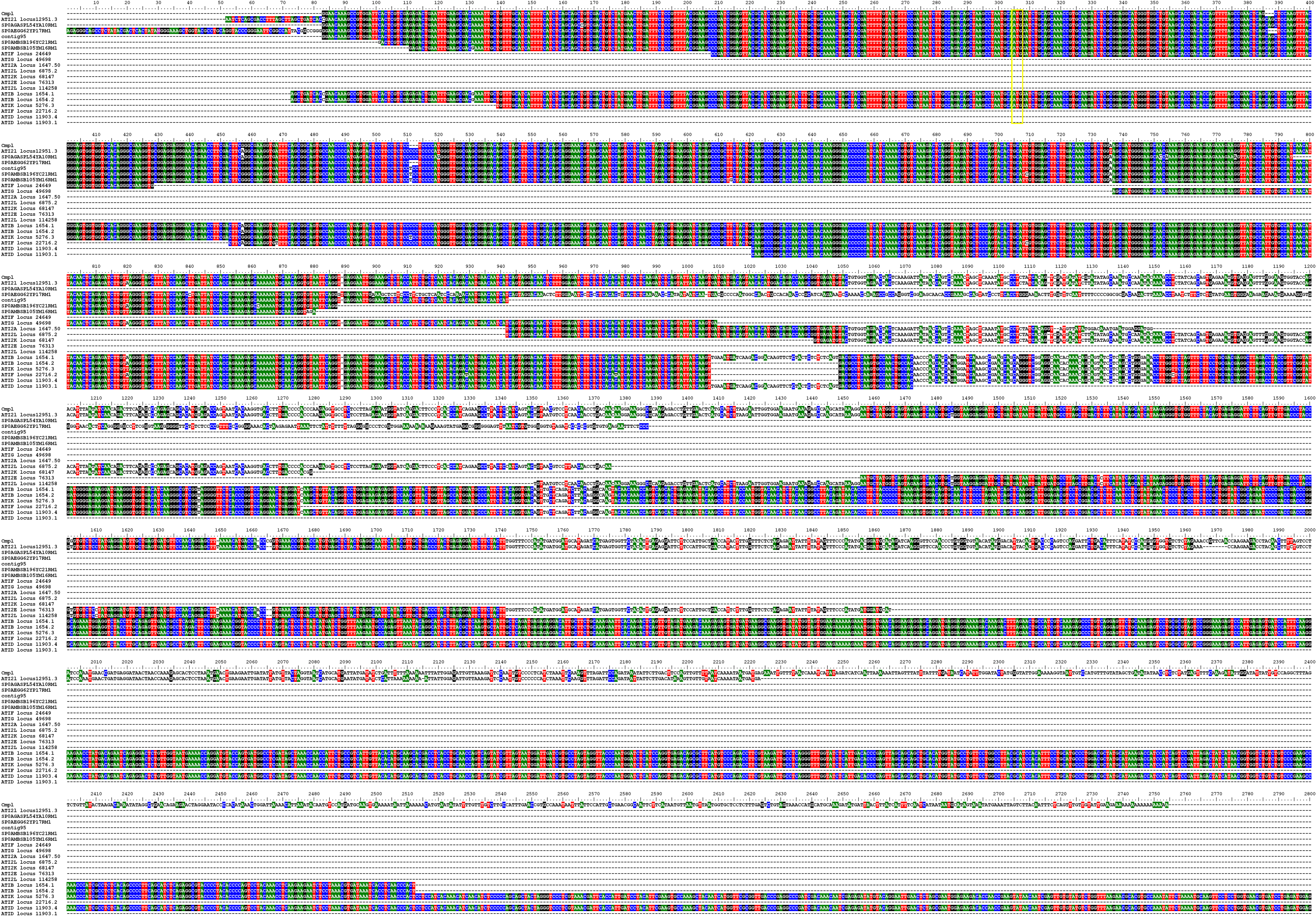

Supplement: Figure S1 — Nucleotide sequence multiple alignment of the Cmpl full cDNA and several ESTs retrieved from the P. lividus database. Yellow square indicates the translation start codon. (TIF) [file pgen.1003847.s001.tif]

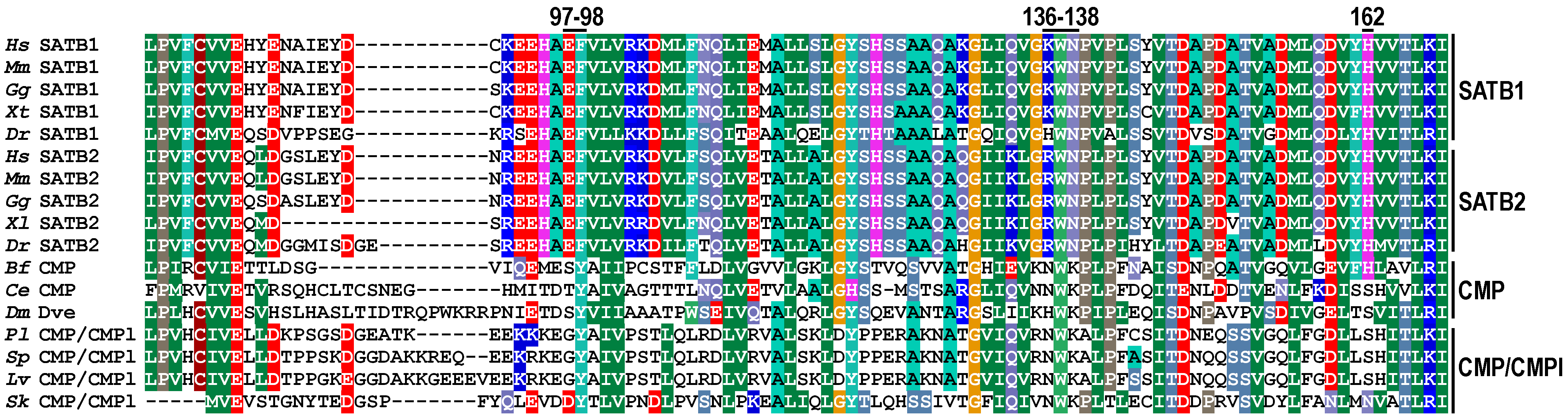

Supplement: Figure S2 — Multiple comparison of the Compass domain of representative SATB, CMP and CMPl family proteins. The position of the residues involved in SATB1 oligomerization are indicated above sequences. Complete taxonomic names and accession numbers of all the sequences used in the alignment are listed in Supplementary Table S1. (TIF) [file pgen.1003847.s002.tif]
