# Supplementary material for: The Compass-like Locus, Exclusive to the Ambulacrarians, Encodes a Chromatin Insulator Binding Protein in the Sea Urchin Embryo
Source: PLoS Genet. 2013 Sep 26;9(9):e1003847. doi: 10.1371/journal.pgen.1003847 (PMC3784565; doi:10.1371/journal.pgen.1003847)
Supplement: Table S1 — Complete taxonomic names and accession numbers of the sequences used in this study. (DOC) [file pgen.1003847.s003.doc]

**Supplementary Table S1**. Complete taxonomic names and accession numbers of the sequences used in this study.

| **Abbreviation** | **Complete name** | **Accession number** | **Type of sequence** | | | **Figure in which sequence was used** |
| --- | --- | --- | --- | --- | --- | --- |
|  |  |  | **Genomic DNA** | **cDNA** | **Protein** |  |
| *Bf* | *Branchiostoma floridae* | XP_002597091.1 (CMP) |  |  | x | Fig. 1, S3 |
| *Ce* | *Caenorhabditis elegans* | NM_001029813.3 (CMP) |  |  | x | Fig. 1, S3 |
| *Cg* | *Crassostrea gigas* | EKC40241.1 (CMP) |  |  | x | Fig. 1 |
| *Dm* | *Drosophila melanogaster* | NM_057894.4 (Dve) |  |  | x | Fig. 1, S3 |
| *Dr* | *Danio rerio* | CAK04309.1 (SATB1) |  |  | x | Fig. S3 |
|  |  | NP_001122004.1 (SATB2) |  |  | x | Fig. 1, S3 |
| *Et* | *Eucidaris tribuloides* | isotig16934 (CMPl) |  | x |  | Fig. 1 |
| *Gg* | *Gallus gallus* | NP_001186573.1 (SATB1) |  |  | x | Fig. 1, S3 |
|  |  | NP_001186039.1 (SATB2) |  |  | x | Fig. 1, S3 |
| *Hs* | *Homo sapiens* | NP_001182399.1 (SATB1) |  |  | x | Fig. 1, S3 |
|  |  | NP_056080.1 (SATB2) |  |  | x | Fig. 1, S3 |
|  |  | NP_004843 (Onecut2) |  |  | x | Fig. 1 |
| *Lv** | *Lythechinus variegatus* | scaffold 1798 | x |  |  | Fig. 2 |
| *Mm* | *Mus musculus* | CAJ18578.1 (SATB1) |  |  | x | Fig. 1, S3 |
|  |  | NP_631885 (SATB2) |  |  | x | Fig. 1, S3 |
| *Pl* | *Paracentrotus lividus* | KF421245 (CMPl) |  | x |  | Fig. 1, S1, S3 |
|  |  | scaffold 01096 | x |  |  | Fig. 1, 2, S3 |
|  |  | scaffold 22924 | x |  |  | Fig. 1, 2, S3 |
|  |  | scaffold 36969 | x |  |  | Fig. 1, 2, S3 |
|  |  | scaffold 51495 | x |  |  | Fig. 1, 2, S3 |
|  |  | scaffold 52488 | x |  |  | Fig. 1, 2, S3 |
|  |  | contig 00064 | x |  |  | Fig. 1, 2, S3 |
|  |  | contig 08288 | x |  |  | Fig. 1, 2, S3 |
|  |  | contig 09355 | x |  |  | Fig. 1, 2, S3 |
|  |  | contig 60426 | x |  |  | Fig. 1, 2, S3 |
|  |  | contig 62655 | x |  |  | Fig. 1, 2, S3 |
|  |  | contig 82961 | x |  |  | Fig. 1, 2, S3 |
|  |  | contig 100117 | x |  |  | Fig. 1, 2, S3 |
|  |  | contig 120697 | x |  |  | Fig. 1, 2, S3 |
|  |  | contig 149045 | x |  |  | Fig. 1, 2, S3 |
|  |  | contig 170979 | x |  |  | Fig. 1, 2, S3 |
|  |  | contig 206549 | x |  |  | Fig. 1, 2, S3 |
|  |  | contig 242706 | x |  |  | Fig. 1, 2, S3 |
|  |  | contig 308777 | x |  |  | Fig. 1, 2, S3 |
|  |  | contig 350373 | x |  |  | Fig. 1, 2, S3 |
|  |  | ATI2I locus 12951.3 |  | x |  | Fig. S1 |
|  |  | ATI2A locus 1647.50 |  | x |  | Fig. S1 |
|  |  | ATI2K locus68147 |  | x |  | Fig. S1 |
|  |  | ATI2L locus 6875.2 |  | x |  | Fig. S1 |
|  |  | ATI2L locus 114258 |  | x |  | Fig. S1 |
|  |  | ATIF locus 24649 |  | x |  | Fig. S1 |
|  |  | ATIG locus 49698 |  | x |  | Fig. S1 |
|  |  | contig 95 |  | x |  | Fig. S1 |
|  |  | ATIB locus 1654.1 |  | x |  | Fig. S1 |
|  |  | ATIB locus 1654.2 |  | x |  | Fig. S1 |
|  |  | ATID locus 11903.1 |  | x |  | Fig. S1 |
|  |  | ATID locus 11903.4 |  | x |  | Fig. S1 |
|  |  | ATIF locus 22716.2 |  | x |  | Fig. S1 |
|  |  | ATIK locus 5276.3 |  | x |  | Fig. S1 |
|  |  | ATI2E locus 76313 |  | x |  | Fig. S1 |
|  |  | SP0AGASPL54YA10RM1 |  | x |  | Fig. S1 |
|  |  | SP0AMBSB105YM16RM1 |  | x |  | Fig. S1 |
|  |  | SP0AMBSB196YC21RM1 |  | x |  | Fig. S1 |
| *Sf* | *Strongylocentrotus franciscanus* | EMHK5CQ03FWU9W (CMPl) | x |  |  | Fig. 1 |
| *Sk** | *Saccoglossus kowalevskii* | NW_003149779.1  (scaffold 44291) | x |  |  | Fig. 1, 2, S3 |
| *Sp* | *Strongylocentrotus purpuratus* | NW_003577017.1  (scaffold 56) | x |  |  | Fig. 2 |
|  |  | WHL22.589656.2 (CMPl) |  | x |  | Fig. 1, S3 |
|  |  | WHL22.589656.3 (CMP) |  | x |  | Fig. 1, S3 |
|  |  | NP_999824 (HNF6) |  |  | x | Fig. 1 |
| *Ts* | *Trichinella spiralis* | ABIR02000599.1 (CMP) | x |  |  | Fig. 1 |
| *Xl* | *Xenopus laevis* | AEJ84495 (SATB2) |  |  | x | Fig. 1, S3 |
| *Xt* | *Xenopus tropicalis* | NP_001093671.1 (SATB1) |  |  | x | Fig. 1, S3 |

*CMPl and CMP protein sequences for these organisms were deduced from genomic scaffold sequences.
