# Supplementary material for: The Compass-like Locus, Exclusive to the Ambulacrarians, Encodes a Chromatin Insulator Binding Protein in the Sea Urchin Embryo
Source: PLoS Genet. 2013 Sep 26;9(9):e1003847. doi: 10.1371/journal.pgen.1003847 (PMC3784565; doi:10.1371/journal.pgen.1003847)
Supplement: Table S2 — List of gene-specific oligonucleotides used in the qPCR analyses. (DOC) [file pgen.1003847.s004.doc]

**Supplementary Table S2**. List of gene-specific oligonucleotides used in the qPCR analyses.

| Target gene | Forward (F) and Reverse (R) sequences (5’ to 3’) | Length | Amplicon size (bp) | Figures in which oligonucleotide was used |
| --- | --- | --- | --- | --- |
|  |  |  |  |  |
| *Cmpl* | F: GCTCTACCATTCTGCTCAATC  R: ACCACAGTACATCTCACCGC | 21  20 | 120 | Fig. 2D |
| *Cmp* | F: GCTCTACCATTCTGCTCAATC  R: CCCTGCTGCTTCGGCTTTG | 21  19 | 166 | Fig. 2D |
| *Sns5* | F: CGGCAAATCAAGCTAAAGGTT  R: GCGTCTGAGTTGTAGTTGCAG | 21  21 | 156 | Fig. 3B |
| *Hbox12* | F: GGAGAGAAGTTGTGAGAGAGC  R: AGGCCTATTATGATTAATCTCAT | 21  23 | 110 | Suppl. Fig. 2 |
| *Otp* | F: GCCTGTACTCATTCAACCAAT  R: CGAAGCCCGTTGGAGTTTTG | 21  20 | 127 | Suppl. Fig. 2 |
| *Gfp* | F: AGGGCTATGTGCAGGAGAGA  R: CTTGTGGCCGAGAATGTTTC | 20  20 | 152 | Fig. 3E |
| *H3* | F: CGTCACCCTCGTTTGATTCACTGAT  R: CGAATCTCTCTCAGTGCGAC | 25  20 | 205 | Fig. 4B-E |
| *H2A* | F: CGCTTCGTCCATTACTTCGCTTCG  R: ACCGCCGCCGACCCTCTTTG | 24  20 | 178 | Fig. 4B-E |
| *H1* | F: TACGAAGGTGACTACTAAGAAGC  R: AAATGTGCCTTGATGTAGTTCGC | 23  23 | 116 | Fig. 4B-E |
| *mbf1*^§^ | F: ATGACACAGCCTGGAGCT  R: TACCAAGGAAGTGGGTGT | 18  18 | 102 | Fig. 2D, 3E, 4B-E |
| *cit-ox*^§^ | F: GTTGGGGTTAATCTAACATTCTTC  R: GAGGGTATAGGCATCTGGATAG | 24  22 | 93 | Fig. 2D, 3E, 4B-E |

^§^A *cytochrome-oxidase* (*cyt-ox*) or the *H2A* histone enhancher binding factor (*mbf1*) mRNA were used to normalize data.
